# *Trichoderma asperellum* Suppresses Gray Mold Caused by *Botrytis cinerea* and Enhances Disease Resistance of Blueberry

**DOI:** 10.3390/jof12070515

**Published:** 2026-07-14

**Authors:** Yanxia She, Xiaotong Song, Tingzhen Wang, Yujia Li, Huiyan Zheng, Shuyuan Wang, Yutong Liu, Fei Gao, Xin Lou, Yuejia Dang

**Affiliations:** 1 School of Life and Health, Dalian University, Dalian 116622, China; sheyanxia@s.dlu.edu.cn (Y.S.); songxiaotong@s.dlu.edu.cn (X.S.); wangtingzhen@s.dlu.edu.cn (T.W.); liyujia@s.dlu.edu.cn (Y.L.); zhenghuiyan@s.dlu.edu.cn (H.Z.); wangshuyuan@s.dlu.edu.cn (S.W.); liuyutong@s.dlu.edu.cn (Y.L.); gaofei@dlu.edu.cn (F.G.); 2 Institute of Modern Agriculture Research, Dalian University, Dalian 116622, China

**Keywords:** *Trichoderma asperellum*, *Botrytis cinerea*, antagonism, transcriptomic analysis, metabolomic analysis, biological control

## Abstract

With growing global recognition of the nutritional value of blueberries, their cultivation has expanded significantly across various regions. However, gray mold disease, caused by *Botrytis cinerea*, remains a major challenge in postharvest storage and transportation. In this study, two strains—*Trichoderma asperellum* BBR–A and *B. cinerea* BC7–1—were isolated and identified from the blueberry rhizosphere and diseased leaves. Co-culture assays demonstrated that BBR–A exhibits potent antagonistic activity, rapidly suppressing the growth of the pathogen BC7–1 through mycoparasitic behaviors, including hyphal coiling, penetration, and degradation. Transcriptomic analysis revealed that upon interaction with BC7–1, BBR–A undergoes extensive reprogramming of carbon metabolism, with coordinated upregulation of genes encoding degradative enzymes, transporters, and stress-related proteins. Congo red staining and enzymatic activity assays further confirmed the enhanced secretion of extracellular enzymes and the increased cell wall-degrading capacity of BBR–A. On detached blueberry leaves, seedlings, and fruits, BBR–A treatment significantly delayed lesion expansion and reduced disease severity. Metabolomic profiling further demonstrated that BBR–A induces the marked accumulation of phenolic compounds, polyamines, and other antioxidant- and immunity-related metabolites in blueberry leaves, thereby maintaining host redox homeostasis and enhancing stress resilience. Collectively, these findings indicate that *T. asperellum* BBR–A effectively suppresses *B. cinerea* through multiple mechanisms, including antagonism, mycoparasitism, metabolic regulation, and the induction of plant defense responses, highlighting its strong potential for development as a biocontrol agent against blueberry gray mold.

## 1. Introduction

Gray mold, caused by *Botrytis cinerea*, is one of the most significant post-harvest diseases affecting blueberry production. This fungal pathogen poses a serious threat to the storage, transportation, and marketing of blueberries [1]. Several chemical fungicides, including pyraclostrobin, boscalid, and azoxystrobin, have demonstrated effective control against *B. cinerea* [2]. However, with the advancement of agricultural productivity and the expansion of international trade, the prevalence of certain plant diseases has increased, leading to the extensive use of chemical pesticides. This not only results in environmental pollution but also leaves persistent chemical residues in agroecosystems. Although alternative strategies such as genetic modification have shown some potential for disease management, they may also increase the risk of pathogen resistance development [3]. In contrast, biological control offers an environmentally sustainable approach that can effectively suppress disease occurrence, enhance crop yield, and avoid the adverse effects associated with chemical pesticides. Therefore, establishing a comprehensive evaluation system for antagonistic microorganisms and successfully applying it in disease management practices holds significant research value and practical importance [4].

Blueberry (*Vaccinium* spp.) belongs to the family Ericaceae and is widely appreciated for its refreshing flavor, sweet taste, and rich nutritional value. In recent years, blueberries have been recognized as a “superfruit” and are listed among the five healthiest fruits globally [5]. This fruit not only offers an appealing taste but also contains various bioactive compounds, including organic acids, phenolic compounds, minerals, and vitamins, which exhibit antioxidant, anti-inflammatory, anticarcinogenic, neuroprotective, and vision-improving properties [6].

The genus *Trichoderma* represents one of the most widely applied groups of plant growth-promoting microorganisms in modern agricultural practices. With its diverse beneficial traits, *Trichoderma* has become an essential component of sustainable agriculture [7]. During interactions with pathogenic fungi, *Trichoderma* can detect and respond to specific secreted compounds produced by the pathogens, exhibiting directed growth toward them [8]. Through the secretion of non-volatile antifungal metabolites and direct mycoparasitism, *Trichoderma* effectively suppresses a wide range of plant pathogenic fungi and oomycetes, highlighting its potential as an alternative to chemical fungicides [9,10]. The biocontrol mechanisms of *Trichoderma* primarily include: (1) the production of hydrolytic enzymes, antibiotics, various bioactive metabolites, and volatile organic compounds [10,11]. Transcriptomic studies have shown that *T. asperellum* upregulates genes related to heat shock proteins and membrane transport during interactions with pathogens, which are involved in protein homeostasis, stress adaptation, and nutrient transport, thereby supporting its colonization and interaction [12,13]; (2) competition for nutrients and ecological niches, where rapid growth provides a significant advantage in securing space and resources [9,14]; (3) mycoparasitism, characterized by the wrapping of hyphae around pathogen hyphae, leading to their lysis and cytoplasmic leakage [15]; and (4) activation of plant defense-related genes and induction of systemic resistance [8,16]. This is manifested by the accumulation of phenolic and flavonoid compounds, increased activity of polyphenol oxidase (PPO) and peroxidase (POD), and elevated total phenol content, which trigger systemic defense responses [17]. Furthermore, it promoted lignification of the cell wall and the activation of defense-related gene expression [18].

In addition, *Trichoderma* species enhance plant tolerance to both biotic and abiotic stresses, including heavy metals, salinity, drought, and low temperature [19,20]. By inducing biochemical changes in carbohydrate, amino acid, organic acid, and lipid metabolism, *Trichoderma* promotes plant growth [19]. Furthermore, it enhances crop yield and growth performance through the production of compounds that stimulate cell division and plant hormone synthesis, solubilization of phosphorus and iron, and the establishment of a balanced plant hormone system [21,22]. In summary, *Trichoderma* plays a crucial and multifaceted role in promoting plant growth, controlling diseases, and enhancing environmental adaptability through a combination of direct and indirect mechanisms.

In this study, a strain of *T. asperellum* was isolated and identified from the roots of blueberry plants, while a strain of *B. cinerea* was isolated and identified from blueberry leaves affected by gray mold disease. In dual-culture experiments, it was observed that *T. asperellum* effectively inhibited the mycelial growth of *B. cinerea* and exhibited clear mycoparasitic behavior. Additionally, transcriptomic analysis combined with proteome-metabolome integration was conducted to elucidate the antifungal mechanisms of *T. asperellum* and its molecular basis for enhancing blueberry disease resistance. The findings of this study aim to provide novel strategies for the sustainable development of blueberry production through the application of *T. asperellum* as a biocontrol agent.

## 2. Materials and Methods

### 2.1. Plant Pathogen and Plant Materials

*T. asperellum* BBR–A was isolated from blueberry root-associated soil using the tissue isolation method; detailed methods are shown in Baiyeeet et al. [23] and deposited in the China Center for Type Culture Collection (CCTCC NO: M 20251239). *B. cinerea* BC7–1 was isolated from blueberry plants exhibiting typical gray mold symptoms using the same tissue isolation method; detailed methods are shown in Chen et al. [24]. The blueberry plants and seedlings used in this study were the cultivar ‘Eureka Sunrise’. All experimental procedures involving plants were performed in accordance with applicable national and local laws and regulations.

### 2.2. Media and Culture Conditions

The PDA medium consisted of potato extract powder (12.0 g/L), glucose (20.0 g/L), and agar (14.0 g/L), with pH adjusted to 5.6 ± 0.2.

The CMC medium consisted of sodium carboxymethyl cellulose (CMC-Na, 10.0 g/L), ammonium sulfate ((NH_4_)_2_SO_4_, 4.0 g/L), dipotassium hydrogen phosphate (K_2_HPO_4_, 2.0 g/L), magnesium sulfate heptahydrate (MgSO_4_·7H_2_O, 0.5 g/L), peptone (10.0 g/L), and beef extract (5.0 g/L), dissolved in distilled water to a final volume of 1 L.

The colloidal chitin medium consisted of ammonium nitrate (NH_4_NO_3_, 3.0 g/L), potassium dihydrogen phosphate (KH_2_PO_4_, 2.0 g/L), magnesium sulfate heptahydrate (MgSO_4_·7H_2_O, 0.6 g/L), ferrous sulfate heptahydrate (FeSO_4_·7H_2_O, 0.1 g/L), and colloidal chitin (5.0 g/L), dissolved in distilled water to a final volume of 1 L.

### 2.3. In Vitro Biocontrol of T. asperellum BBR–A Against B. cinerea BC7–1

A dual-culture assay was conducted on PDA plates. One half of each plate was inoculated with *T. asperellum* BBR–A and the other half with *B. cinerea* BC7–1. As a control, BC7–1 was inoculated alone on PDA plates under identical conditions. Three biological replicates were included, and the experiment was independently repeated three times. Colony diameters of BC7–1 in both control and treatment groups were measured every 24 h using the cross-measurement method for four consecutive days, and mean values were recorded.

For light microscopy observation (Nexcope NE620, Ningbo, China), sterile coverslips were placed at the interaction zone between the two fungi on PDA plates. Coverslips were removed at three time points: when mycelia first contacted each other, 1 day after contact, and 2 days after contact. Samples were mounted on slides and observed under a light microscope to document mycoparasitic interactions.

For scanning electron microscopy (Hitachi S–4800, Tokyo, Japan), a similar dual-culture setup was used, with sterile silicon wafers placed in the confrontation zone. After 1 day of mycelia contact on the silicon wafers, samples were collected, fixed, dehydrated, dried, and gold-coated. Mycelia morphology and surface structural changes were then examined and recorded using SEM.

### 2.4. Transcriptome Analysis

*T. asperellum* BBR–A and *B. cinerea* BC7–1 were co-cultured on the same PDA plate. When their mycelia intersected on the coverslip, the mycelia of BBR–A at the confrontation edge were collected. As a control, BBR–A was cultured alone on PDA for the same duration and sampled accordingly. All samples were subjected to transcriptome sequencing. Sequencing and preliminary data processing were performed by Beijing Novogene Bioinformatics Technology Co., Ltd. (Beijing, China). Results were visualized using appropriate bioinformatics tools.

### 2.5. Quantitative Real-Time PCR (qRT-PCR)

Total RNA was extracted from 7-day-old fungal mycelia grown on PDA plates using the Spin Column Fungal Total RNA Purification Kit (Sangon Biotech, Shanghai, China). For cDNA synthesis, 2 μg of total RNA per sample was reverse-transcribed using All-in-One 5× RT MasterMix (Applied Biological Materials Inc, Richmond, BC, Canada). Quantitative real-time PCR was performed using the CFX96 Optical Reaction Module (Bio-Rad, Singapore) with BlasTaq™ 2 × qPCR MasterMix (Applied Biological Materials Inc, Richmond, BC, Canada). Relative gene expression levels were calculated using the 2^−ΔΔCq^ method (Cq = Cq_gene_ − Cq_actin_). The actin gene (*TrAFT101_008842*) of *T. asperellum* was used as the internal reference for normalization. Each sample was analyzed in three technical replicates. Primer sequences (Sangon Biotech, Shanghai, China) used for qRT-PCR are listed in Table S1.

### 2.6. Enzyme Activity Determination of T. asperellum BBR–A Before and After Confrontation with B. cinerea BC7–1

*T. asperellum* BBR–A and *B. cinerea* BC7–1 were co-cultured on PDA plates. When mycelia intersected on the coverslip, confrontation-edge mycelia of BBR–A were collected. As a control, BBR–A was cultured alone under identical conditions. After incubation at 28 °C for 3 days, two mycelial plugs from both treatment and control groups were transferred into cellulase-inducing and chitinase-inducing media, respectively. The plugs were inoculated into 250 mL Erlenmeyer flasks containing 40 mL fermentation medium and incubated at 28 °C with shaking at 180 r/min for 4 days. Cultures were centrifuged at 4 °C and 5000 r/min for 10 min, and the supernatants were collected as crude enzyme extracts. Chitinase activity was determined according to the method described by Preety [25] with slight modifications, and cellulase activity was measured according to Sengupta S [26] with slight modifications.

### 2.7. Control Effects of T. asperellum BBR–A Against B. cinerea BC7–1 Causing Gray Mold of Blueberry

In both detached and in vivo conditions, blueberry leaves of similar size and growth status were selected and divided into four groups. Four treatments were set up in this study: pathogen-only (*B. cinerea* BC7–1), biocontrol-only (*T. asperellum* BBR–A), combined inoculation (*T. asperellum* BBR–A pretreatment followed by *B. cinerea* BC7–1 infection), and sterile water control. The pathogen-only and biocontrol-only groups were individually sprayed with the corresponding conidia suspensions; the combined group was pretreated with BBR–A conidia suspension 2 days before inoculation with BC7–1; the control received only sterile water. The conidia concentration in all treatments was adjusted to 1 × 10^6^ conidia/mL. Each treatment included three biological replicates, and the experiment was repeated three times. Similarly, under detached conditions, blueberry fruits of uniform size were selected, divided into four groups, and subjected to the same treatment procedures described above.

### 2.8. Metabolomics Analysis

Blueberry leaves were sampled after being sprayed with a conidial suspension of *T. asperellum* or water, with more than six biological replicates per treatment. At sampling, the leaves were quickly cut and immediately immersed in liquid nitrogen to quench metabolism, then transferred to an ultra-low-temperature freezer at −80 °C for storage. All samples were transported to Hangzhou Jingjie Biotechnology Co., Ltd (Hangzhou, China). Before analysis, the samples were ground into fine powder under liquid nitrogen. An appropriate amount of powder was mixed with pre-chilled 80% methanol in water (methanol: water = 80:20, *v*/*v*), vortexed, ultrasonicated in an ice bath, and centrifuged at low temperature. The supernatant was filtered through a 0.22 μm membrane before LC-MS analysis. Chromatographic separation was performed on a reversed-phase C18 column (Thermo Scientific Dionex UltiMate 3000 UHPLC system, Waltham, MA, USA) at 40 °C, with mobile phase A consisting of 0.1% formic acid in water and mobile phase B consisting of 0.1% formic acid in acetonitrile, using gradient elution at a flow rate of 0.3 mL/min. Mass spectrometry was conducted using a high-resolution mass spectrometer (Thermo Fisher Scientific, Waltham, MA, USA) equipped with an ESI source, and data were acquired in both positive and negative ion modes over a scan range of *m*/*z* 70–1050. Metabolites were identified based on retention time, accurate mass-to-charge ratio, and MS/MS fragment information. The acquired data were preprocessed by peak alignment, noise filtering, and normalization before being used for multivariate statistical analysis.

### 2.9. DAB Staining and Measurement of H_2_O_2_ Content

Detached leaves were treated with *T. asperellum* BBR–A spore suspension for 1 day, while the control group was treated with sterile water. Detailed methods were described in Daudi, A [27]. Blueberry leaves were sprayed with BBR–A conidia suspension at a concentration of 1 × 10^6^ conidia/mL, while the control group (CK) was sprayed with distilled water. Leaf samples were collected one day after treatment. Weigh 0.2 g of leaf tissue and homogenize with 2 mL of cold acetone. Centrifuge at 12,000 rpm and 4 °C for 20 min. Transfer the supernatant into a 2 mL centrifuge tube, add 100 μL of 20% titanium tetrachloride (TiCl_4_) in concentrated HCl, then dropwise add 700 μL of 20% ammonia water. Centrifuge at 10,000 rpm and 4 °C for 5 min, discard the supernatant. Suspend and wash the precipitate with cold acetone at least 5 times to reduce pigment interference. Dissolve the precipitate with 1 mol/L sulfuric acid and make up the volume to 10 mL, then measure the absorbance.

### 2.10. Measurement of POD Content

Cut 0.1 g of leaf tissue and place it into a mortar kept in an ice bath. Add 2 mL of a 20 mmol/L KH_2_PO_4_ solution and grind the leaf tissue to a homogenate. Then rinse the mortar with 1 mL of 20 mmol/L KH_2_PO_4_ solution. Transfer the entire homogenate into centrifuge tubes and centrifuge at 4000 r/min for 10 min. Collect the supernatant and store it in a 4 °C refrigerator (Haier, Qingdao, China) for later use. The activities of POD were assayed in accordance with Maehly’s method [28].

### 2.11. Data Statistics and Analysis

All experiments were conducted with at least three biological replicates. Colony diameters were measured using ImageJ 1.54g Statistical analyses of germination rates and relative gene expression levels were performed using Prime 10.6. Data are presented as mean ± standard deviation (SD), and error bars represent SD. One-way ANOVA was used for statistical comparisons. Statistical significance was defined as follows: ns, not significant (*p* > 0.05); *, significant (*p* < 0.05); **, highly significant (*p* < 0.01); ***, extremely significant (*p* < 0.001).

## 3. Results

### 3.1. Growth, Morphological Characteristics, and Identification of Fungal Strains

**Figure 1:**
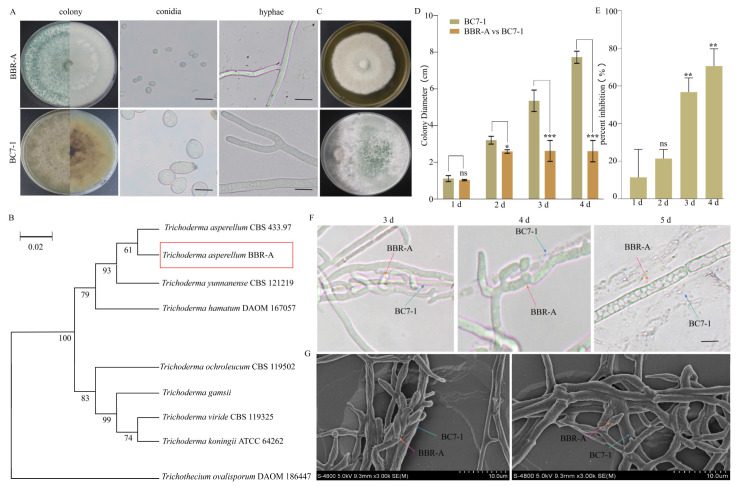
Morphological observation of *T. asperellum* BBR–A, *B. cinerea* BC7–1, and analysis of their confrontation interaction (**A**) Colony, conidia, and hyphae of BBR–A and BC7–1 growth on PDA, with light microscopy. Scale bar, 15 μm. (**B**) Phylogenetic tree of *T. asperellum* BBR–A. Evolutionary history was reconstructed using the Neighbor-Joining method. Scale bar, 0.02 substitutions per site. (**C**) Dual culture of BBR–A and BC7–1 on PDA. (**D**) Differences in colony size of BC7–1. (**E**) Inhibition rate of colony diameter of BC7–1 under dual culture. (**F**) Microscopic observations of the interaction at 3, 4, and 5 days after confrontation. Scale bar, 15 μm. (**G**) Electron microscopy observations at 3 days after confrontation. Error bars represent mean ± SD from three replicates. ns, *p* > 0.05; * *p* < 0.05, ** *p* < 0.01, *** *p* < 0.001.

Fungal strains were isolated from blueberry roots and gray mold-diseased leaves, respectively. Based on ITS sequencing and BLAST (https://blast.ncbi.nlm.nih.gov/Blast.cgi, accessed on 1 June 2026) analysis (Figure S1), the isolates were identified and designated as *T. asperellum* BBR–A and *B. cinerea* BC7–1. Morphological observations on PDA plates revealed that the colony of BBR–A grew rapidly, appearing circular with entire margins. Initially white, the colony gradually turned light green to dark green. The surface was powdery, and the texture was loose, with the reverse side ranging from light green to colorless. Microscopic examination showed hyphae that were transparent, septate, and abundantly branched, bearing spherical or ellipsoidal conidia (Figure 1A). In contrast, the colony of BC7–1 was grayish-white to gray in color with a velvety texture and exhibited rapid growth. After prolonged cultivation, a large number of gray, powdery conidia formed on the surface. Microscopic analysis revealed transparent, septate, and branched hyphae. The conidiophores were erect, with dichotomously branched apices. Conidia were ellipsoidal to ovoid, hyaline or lightly pigmented, and arranged in chains (Figure 1A). Phylogenetic analysis based on ITS sequences demonstrated that BBR–A clustered with the type strain *T. asperellum* CBS 433.97, supported by a bootstrap value of 61 (Figure 1B), indicating a close phylogenetic relationship and further confirming the taxonomic identity of *T. asperellum* BBR–A.

### 3.2. Inhibitory Effect of T. asperellum BBR–A on B. cinerea BC7–1 and Its Mycoparasitic Activity

In the PDA plate confrontation assay, *T. asperellum* BBR–A exhibited a significant antagonistic effect against *B. cinerea* BC7–1. At the colony level, the growth rate of BBR–A was significantly faster than that of BC7–1, and it contacted and covered the edge of the pathogen colony by day 3, forming a distinct inhibition zone (Figure 1C). Statistical analysis showed that compared with the control group (BC7–1 cultured alone), the colony diameter of BC7–1 in the confrontation group was significantly reduced on day 2, and the inhibitory effect gradually increased over time. By day 3 and day 4, the differences reached a highly significant level (*p* < 0.001) (Figure 1D), indicating that BBR–A possesses strong antifungal activity. The inhibition rate of bacterial colonies reached a highly significant level on the third day and exceeded 70% on the fourth day (Figure 1E). Microscopic observation further revealed typical mycoparasitic behavior. On day 3 of the hyphal confrontation, BBR–A hyphae actively wrapped the hyphae of BC7–1, forming a tight spiral structure, which is a characteristic of early parasitic interaction. On day 4, BBR–A hyphae penetrated the cell wall of the pathogen, accompanied by deformation, collapse, and cytoplasmic leakage of BC7–1 hyphae. By day 5, most BC7–1 hyphae were severely degraded and lysed, with loss of cell wall integrity (Figure 1F). Additionally, scanning electron microscopy on day 3 confirmed the wrapping and close adhesion of BBR–A hyphae to BC7–1 hyphae (Figure 1G), indicating structural changes at the interaction interface.

Overall, the antagonistic process of BBR–A against BC7–1 followed a clear temporal sequence: recognition, wrapping, penetration, and degradation. This process represents the typical mycoparasitic behavior of the genus *Trichoderma*. Previous studies have shown that *T. asperellum* is also capable of penetrating the roots of plant seedlings and colonizing the epidermis and outer cortex of the root. These interactions can induce systemic resistance in the host plant against pathogens [29].

### 3.3. Transcriptomic Analysis of T. asperellum BBR–A in Inhibiting B. cinerea BC7–1

**Figure 2:**
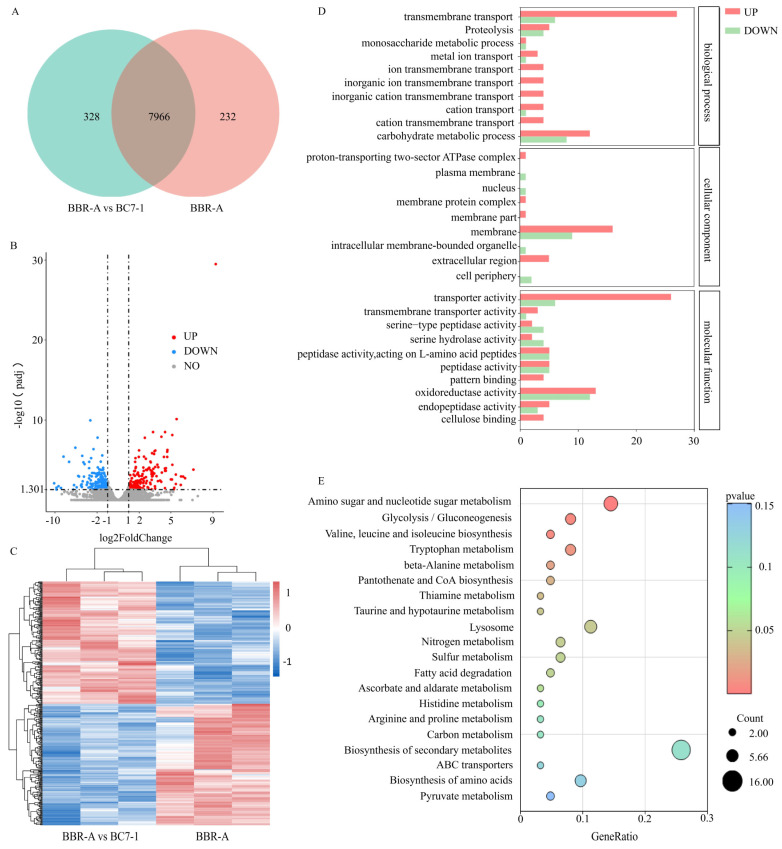
Transcriptomic analysis of *T. asperellum* BBR–A in response to *B. cinerea* BC7–1stress. (**A**) Venn diagram of gene expression between BBR–A and BBR–A vs. BC7–1. (**B**) Volcano plot of differentially expressed genes (DEGs) between BBR–A and BBR–A vs. BC7–1. (**C**) Hierarchical clustering heatmap of DEGs. (**D**) GO enrichment analysis of DEGs, categorized into biological process, cellular component, and molecular function. (**E**) KEGG pathway enrichment scatter plot.

To elucidate the antifungal mechanism of *T. asperellum* BBR–A against *B. cinerea* BC7–1, this study compared the transcriptomes of BBR–A mycelia co-cultured with BC7–1 (treatment group) and normally growing BBR–A mycelia (control group). A transcriptomic comparison was conducted between the two groups. To further validate the reliability of the RNA-Seq results, a subset of differentially expressed genes (DEGs) was selected for qRT-PCR analysis. qPCR validation was performed to verify the RNA-Seq results (Figure S2). The results showed that the expression of relevant genes differed significantly in the treatment group (*p* < 0.05), and their expression patterns were consistent with those obtained from the transcriptomic data, confirming the accuracy of the RNA–Seq results. A total of 7966 genes were expressed across all samples (Figure 2A). Volcano plot analysis revealed a large number of differentially expressed genes (DEGs), including 178 significantly upregulated genes and 176 significantly downregulated genes (Figure 2B). The overall heatmap further confirmed that the transcriptome profiles of BBR–A exhibited significant differences before and after co-cultivation with BC7–1 (Figure 2C).

GO functional enrichment analysis revealed the main functional categories of the differentially expressed genes (Figure 2D). In the biological process category, the DEGs were significantly enriched in transmembrane transport and carbohydrate metabolism, including transmembrane transport (27 upregulated, 6 downregulated), proteolysis (5 upregulated, 4 downregulated), and carbohydrate metabolic processes (12 upregulated, 8 downregulated). In the cellular component category, the DEGs were mainly enriched in membrane-related structures, including membrane components (16 upregulated, 9 downregulated) and extracellular regions (5 upregulated), suggesting that membrane proteins and transmembrane structures may play a key role in the antifungal process. In the molecular function category, the DEGs were significantly enriched in transporter activity (26 upregulated, 6 downregulated), transmembrane transporter activity (3 upregulated, 1 downregulated), and cellulose binding (4 upregulated), indicating that BBR–A may enhance its ability to transport substances across membranes and utilize carbon sources during the response to pathogen stress. Additionally, oxidoreductase activity was significantly enriched (13 upregulated, 12 downregulated), with related genes involved in reactive oxygen species scavenging, antioxidant defense, and carbohydrate metabolism. Some oxidative-reduction-related metabolic activities were suppressed, reflecting oxidative damage and energy metabolism disturbances caused by pathogen stress.

KEGG pathway enrichment analysis, visualized through a bubble plot, showed the significantly enriched metabolic pathways of the differentially expressed genes (Figure 2E). The pathways that were commonly enriched and showed significant differences between the treatment and control groups included amino sugar and nucleotide sugar metabolism, glycolysis/gluconeogenesis, valine/leucine/isoleucine biosynthesis, and tryptophan metabolism. Among these, the amino sugar and nucleotide sugar metabolism and glycolysis/gluconeogenesis pathways had higher gene ratios and stronger enrichment significance, indicating that the carbon metabolism network was significantly reprogrammed during the antifungal process of BBR–A.

In summary, *T. asperellum* BBR–A undergoes significant changes in carbon source utilization, energy metabolism, and transmembrane substance transport during the inhibition of B. cinerea BC7–1, suggesting that the strain enhances its environmental adaptability and biological defense potential through metabolic reprogramming.

### 3.4. T. asperellum Enhances Extracellular Enzyme Secretion and Pathogen Cell Wall Degradation Ability by Synergistically Upregulating Degradative Enzymes, Transporters, and Stress-Related Genes During Antagonistic Interactions

**Figure 3:**
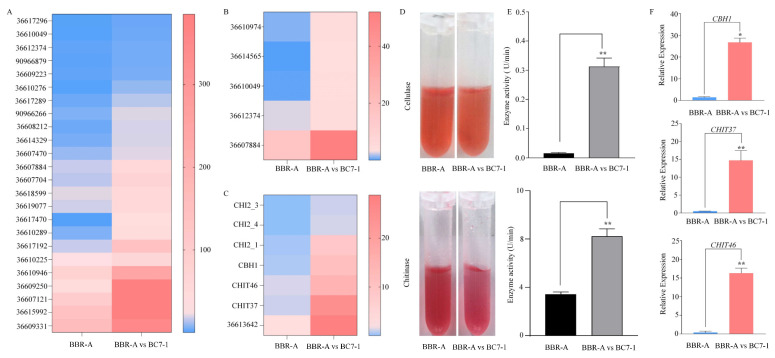
Expression heatmaps of key functional genes and enzyme activity of *T. asperellum* BBR–A under *B. cinerea* BC7–1 stress. (**A**) Heatmap of transmembrane protein–encoding genes. (**B**) Heatmap of heat shock protein (HSP) and transporter protein–encoding genes. (**C**) Heatmap of glycoside hydrolase family genes. (**D**) Comparison of Congo red staining of chitin and cellulose after treatment with crude enzyme solution before and after confrontation. (**E**) Quantitative analysis of enzyme activity. (**F**) Relative expression levels of key genes validated by qPCR. Error bars represent mean ± SD from three replicates. * *p* < 0.05, ** *p* < 0.01.

Previous studies have shown that the enhancement of membrane activity can significantly improve antimicrobial efficacy [30]. In this study, several functionally related genes associated with antagonism were significantly upregulated under the confrontation conditions. First, multiple genes related to transmembrane proteins showed a marked upregulation trend (Figure 3A), particularly those involved in substance transmembrane transport and membrane integration, suggesting that the strain enhanced its nutrient uptake, metabolite secretion, and signal transduction capabilities after being stimulated, thereby improving its adaptability and competitive advantage in the environment.

Heat shock proteins serve as important antibacterial targets [31], capable of inhibiting hyphal growth and reducing the virulence of pathogens. The ABC transporter-mediated efflux mechanism is highly conserved in biocontrol fungi and represents one of the key mechanisms underlying their biocontrol activity [32]. Additionally, changes in the expression of ABC transporter-related genes are noteworthy (Figure 3B). Previous studies have demonstrated that ABC transporters can facilitate the uptake of chitobiose (GlcNAc_2_) into the cell, which in turn induces the expression of the chitinase gene *ECH42*. When the function of this transporter is disrupted, the antifungal activity of *Trichoderma* is significantly reduced [33]. This mechanism not only helps the biocontrol fungus resist host toxins but also promotes the secretion of antimicrobial substances [34]. In this study, the expression levels of heat shock protein and transporter-related genes were significantly increased following confrontation treatment (Figure 3B), indicating that the strain activated a stress protection mechanism during interaction with the pathogen to maintain protein homeostasis and cellular structural stability.

Research has demonstrated that chitinases, cellulases, and other polysaccharide-degrading enzymes play a pivotal role in the mycoparasitic interaction between *Trichoderma* spp. and phytopathogenic fungi by mediating the hydrolysis of structural polysaccharides in the fungal cell wall [35]. Transcriptomic analysis revealed that the expression levels of genes encoding glycoside hydrolases were significantly upregulated (Figure 3C), with notable increases in the expression of *Chit46*, *Chit37*, and *Chi2* family chitinase genes, and *CBH1* showed an upregulation of nearly 20-fold (Figure 3F). Therefore, the synergistic upregulation of genes encoding glycoside hydrolases, transporters, transmembrane proteins, and heat shock proteins reflects the strain’s enhanced ability to degrade the cell wall, improve the efficiency of substance transport, and respond to stress at the transcriptional level, providing important molecular evidence for its antagonistic and biocontrol functions.

To further verify the changes in the activity of relevant hydrolases in the confrontation treatment group, we conducted a Congo red staining experiment and observed that the confrontation culture group exhibited faster and more pronounced decolorization in media containing cellulose or chitin (Figure 3D), indicating a stronger ability to degrade substrates. Enzymatic activity assays were performed, and standard curves were generated (Figure S3A,B). The results showed that chitinase and cellulase activities in the confrontation group were significantly higher compared to the control group (Figure 3E). Meanwhile, qPCR analysis confirmed that the expression levels of corresponding genes were significantly upregulated by approximately 15–30-fold (Figure 3F), consistent with the enzymatic activity changes. These results indicate that *T. asperellum* BBR–A enhances the secretion of extracellular enzymes through transcriptional regulation, thereby improving its antagonistic ability.

### 3.5. T. asperellum BBR–A Effectively Suppresses the Pathogenicity of B. cinerea BC7–1

**Figure 4:**
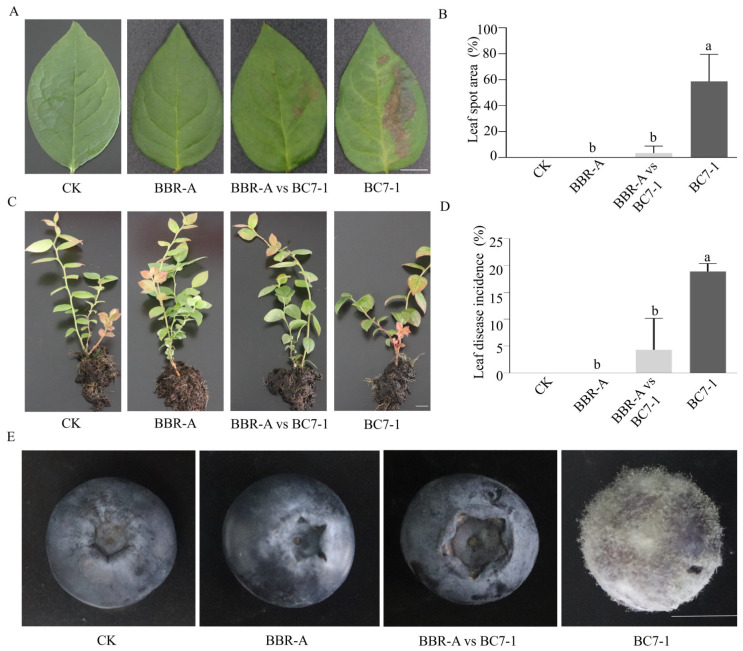
Biocontrol efficacy of *T. asperellum* BBR–A against blueberry gray mold. (**A**) Phenotype observation of detached leaves after spraying with conidial suspension. (**B**) Statistical analysis of leaf spot area on detached leaves after spraying with conidial suspension. (**C**) Phenotype observation of live seedlings after spraying with conidial suspension. (**D**) Statistical analysis of leaf disease incidence on live seedlings after spraying with conidial suspension. (**E**) Phenotype observation of blueberry fruits after dripping with conidial suspension. Scale bar, 1 cm. Error bars represent mean ± SD from three replicates. Different letters denote significant differences at *p* < 0.05 (one-way ANOVA, Tukey’s test).

To evaluate the inhibitory effect of *T. asperellum* BBR–A on the pathogenicity of *B. cinerea* BC7–1, this study conducted detached leaf assays, whole-plant (seedling) inoculation assays, and blueberry fruit infection experiments. The results showed that in the detached leaf assay, no disease symptoms were observed on leaves treated only with BBR–A conidia, whereas leaves pre-treated with BBR–A conidia before challenge with BC7–1 conidia exhibited significantly delayed lesion development, smaller lesion areas, and slower expansion; in some cases, only mild localized necrosis was observed. In contrast, leaves inoculated solely with BC7–1 conidia developed visible lesions rapidly, which expanded quickly and formed large, diffuse brown necrotic areas (Figure 4A). Further statistical analysis of lesion area revealed that the mean lesion area on leaves inoculated with BC7–1 alone was significantly larger than that on BBR–A pre-treated leaves (Figure 4B). Similar results were observed in the whole-plant inoculation assay using *Vaccinium* seedlings (Figure 4C), with statistical analysis confirming a significant increase in leaf disease incidence in the BC7–1-only group compared to the BBR–A pre-treated group (Figure 4D). Additionally, in the fruit infection experiment, BBR–A pre-treated fruits showed no disease symptoms throughout the observation period, while fruits inoculated solely with BC7–1 were completely covered by dense, grayish mycelium typical of *B. cinerea* infection (Figure 4E). Collectively, these findings demonstrate that *T. asperellum* BBR–A effectively suppresses the pathogenicity of *B. cinerea* BC7–1 across different host tissues, highlighting its potential as a promising biocontrol agent against gray mold in blueberry and other horticultural crops.

### 3.6. Metabolomic Analysis of Blueberry Leaves Treated with T. asperellum BBR–A

*Trichoderma* species are known to produce a variety of secondary metabolites that play important roles in modulating plant defense mechanisms, inducing the expression of defense-related genes, and enhancing systemic resistance [36]. Previous studies have shown that *T. asperellum* BBR–A can effectively suppress the pathogenicity of *B. cinerea* BC7–1. However, whether BBR–A also enhances the defense and disease resistance of blueberry leaves during this treatment remains to be investigated.

**Figure 5:**
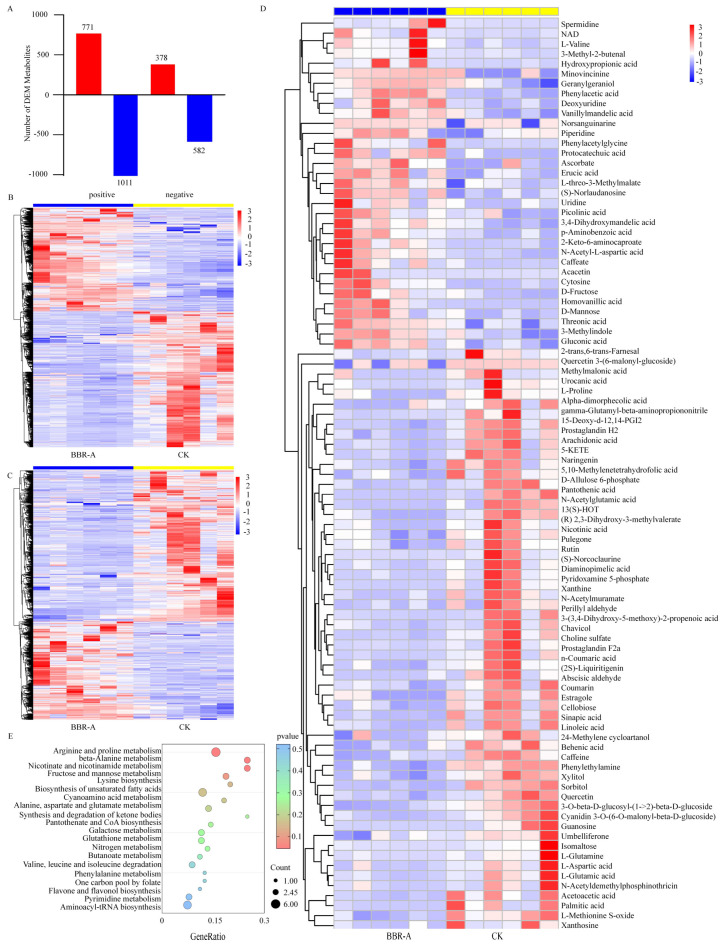
Proteometabolomic profiles of blueberries treated with *T. asperellum* BBR–A. (**A**) Number of up-regulated and down-regulated genes in positive and negative ion modes, with red representing up-regulated genes and blue representing down-regulated genes. (**B**) Heatmaps of total differential metabolites in positive ion mode. (**C**) Heatmaps of total differential metabolites in negative ion mode. (**D**) Heatmap of secondary differential metabolites of proteins. Yellow color stands for BBR–A group, while blue represents the CK control group. (**E**) Bubble plot of KEGG differential enrichment pathways of protein metabolism.

To address this question, we conducted a proteomic and metabolomic analysis of blueberry leaves treated with BBR–A conidia compared to untreated control leaves. The results showed that a total of 2749 significantly different metabolites were identified, with 771 upregulated and 1011 downregulated in cationic metabolism, and 378 upregulated and 582 downregulated in anionic metabolism (Figure 5A). The differential heatmaps of anionic and cationic metabolites clearly indicate that BBR–A treatment significantly altered the metabolic profile of the plant (Figure 5B,C).

Further analysis of secondary metabolites revealed that 92 metabolites showed significant differences, with a pronounced enrichment of immune-associated metabolites. Among these, several metabolites related to plant immunity were significantly upregulated, including phenolic derivatives such as vanillic acid, syringic acid, and protocatechuic acid, as well as polyamine signaling molecules like spermidine and antioxidant compounds such as ascorbic acid (Figure 5D). These compounds are functionally linked to key defense pathways: vanillic acid exhibits broad-spectrum antibacterial activity against foodborne pathogens [37]; syringic acid demonstrates potent antifungal properties [38]; protocatechuic acid has been implicated in walnut resistance to *Xanthomonas arboricola* pv. *juglandis*, a causal agent of bacterial blight [39]; spermidine, a polyamine, has been proven to enhance plant resistance to rice blast disease [40]; and ascorbic acid, on the other hand, participates in complex hormone-mediated signaling networks, regulating plant responses to ozone, pathogens, and senescence [41]. Collectively, the coordinated accumulation of these metabolites contributes to the reinforcement of cellular redox balance, membrane integrity, and subcellular compartmentalization, thereby bolstering host immunity. These metabolic regulatory mechanisms provide crucial molecular insights into the immune response of plants under *T. asperellum* treatment, highlighting the pivotal role of metabolic reprogramming in shaping fungal biocontrol efficacy and plant resilience.

KEGG enrichment analysis further revealed that the top 20 enriched metabolic pathways included arginine and proline metabolism, β-alanine metabolism, nicotinate and nicotinamide metabolism, fructose and mannose metabolism, lysine biosynthesis, unsaturated fatty acid biosynthesis, cyanate amino acid metabolism, alanine, aspartate, and glutamate metabolism, ketone body synthesis and degradation, pantothenate and coenzyme A biosynthesis, and galactose metabolism (Figure 5E). These findings suggest that BBR–A treatment significantly modulates the complex metabolic network of blueberry plants, thereby enhancing their disease resistance.

**Figure 6:**
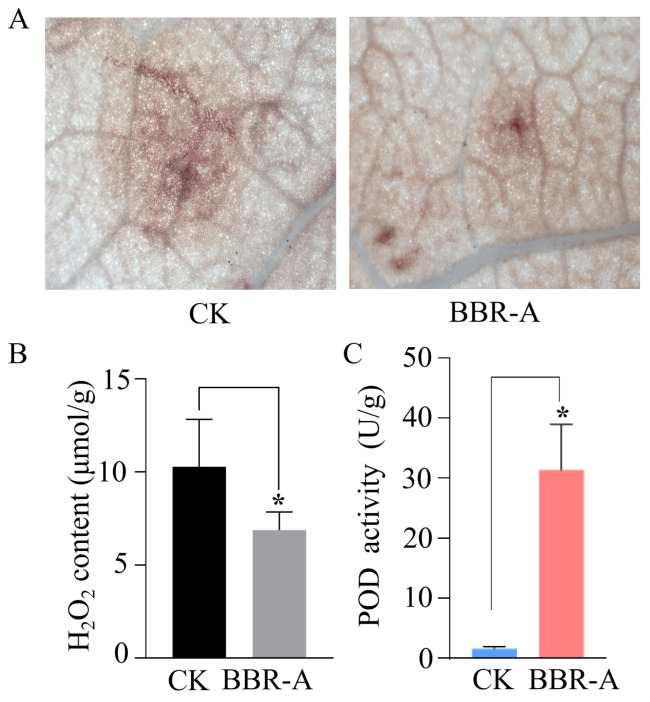
Induction of plant antioxidant responses by *T. asperellum* BBR–A. (**A**) DAB staining of leaves. (**B**) Hydrogen peroxide content. (**C**) Antioxidant enzyme activity in leaves. Error bars represent mean ± SD from three replicates. * *p* < 0.05.

The antioxidant enzymes secreted by Trichoderma into the apoplast of plant cells help alleviate the oxidative burst in plants, thereby reducing the intense defense response mediated by peroxidase and minimizing its harmful effects on the host [42]. To investigate this, we conducted 3,3′-diaminobenzidine (DAB) staining to detect the accumulation of hydrogen peroxide (H_2_O_2_) in blueberry leaves before and after inoculation with *T. asperellum*, and a standard curve was plotted in Figure S3C. Additionally, the activities of key antioxidant enzymes were measured. The results showed that BBR–A treatment significantly reduced H_2_O_2_ accumulation in the leaves compared to the control (CK) (Figure 6A). Quantitative analysis of H_2_O_2_ revealed significant differences among the treatment groups (Figure 6B). Analysis of antioxidant enzyme activity showed that peroxidase (POD) activity was significantly higher in the BBR–A treatment group than in the control (Figure 6C). These findings further indicate that BBR–A enhances disease resistance in blueberries by directly scavenging reactive oxygen species through the accumulation of antioxidant metabolites and by inducing the synthesis and activation of key antioxidant enzymes. 

## 4. Discussion

**Figure 7:**
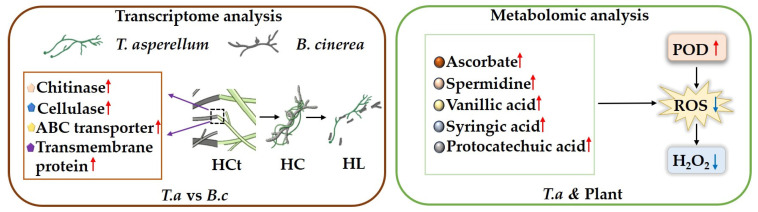
Mechanisms Underlying the Biocontrol and Resistance Induction of *T. asperellum* Against Blueberry Gray Mold. The fungal hyphae model was created using BioGDP.com. [49]. HCt, confrontation culture between *T. asperellum* and *B. cinerea*; HC, hyphal coiling; HL, hyphal lysis; ↑, upregulation; ↓, downregulation.

This study isolated a strain of *T. asperellum* BBR–A from the rhizosphere of blueberry and systematically elucidated its synergistic mechanisms in direct antagonism against phytopathogens and induction of plant resistance through integrated transcriptomic, proteomic, and metabolomic analyses. Figure 7 presents a comprehensive interaction model of *T. asperellum* with *B. cinerea* and blueberry, encompassing key processes such as direct inhibition of the pathogen, activation of host plant immune signaling pathways, and metabolic reprogramming, thereby demonstrating a multi-pathway biocontrol strategy. *Trichoderma* species are known for their broad-spectrum antagonistic activity against various plant pathogens [43,44]. Previous studies have shown that *T. asperellum* exhibits an inhibition rate exceeding 84% against *Lasiodiplodia theobromae*, and up to 100% against several *Colletotrichum* spp. causing agave leaf spot disease. Its inhibitory effect on *Magnaporthiopsis maydis* is closely related to mycoparasitism [45,46,47]. In this study, BBR–A indeed significantly inhibited the mycelial growth of BC7–1 and exhibited a parasitic phenomenon (Figure 2). In addition to Botrytis cinerea, blueberry is also threatened by various pathogenic fungi such as *Cladosporium guizhouense*, *Fusarium annulatum*, and *Neopestalotiopsis surinamensis* [44]. Currently, research on the effect of BBR–A against the pathogens causing blueberry leaf spot and root rot is ongoing. Therefore, further broad-spectrum antifungal studies on BBR–A are warranted, and its potential applications in blueberry production should be fully explored. However, differences in the biological characteristics of different pathogens may influence the dominant mechanisms of *Trichoderma* action. For example, soil-borne *Fusarium* species primarily rely on rhizosphere infection [48], while *B. cinerea* possesses strong saprophytic ability and air-borne transmission. Thus, in the specific system of blueberry gray mold, nutrient competition and stress regulation may play more critical roles. This provides a reasonable biological background for this study focusing on metabolic reprogramming and redox regulation.

In terms of direct antagonism, previous studies have demonstrated that the transmembrane protein Sfp2 of the Sur7 family in *Trichoderma* spp. serves as a key positive regulator in the mycoparasitic process. Heterologous overexpression of Sfp2 significantly enhances the inhibitory activity of the strain against various plant pathogenic fungi [50]. Transcriptomic data from this study revealed significant changes in membrane-related genes during the interaction between BBR–A and BC7–1 (Figure 3A), suggesting that these membrane-localized proteins may play a potential regulatory role in early antagonistic events such as microbial recognition, adhesion, or signal perception. Notably, glycoside hydrolase-related genes were significantly upregulated during the interaction, consistent with previous reports that *T. asperellum* suppresses pathogens by secreting chitinases [51], and similar upregulation of glycoside hydrolase genes has been observed in *T. harzianum* T-22 during the suppression of *Phytophthora capsici* [52]. It is worth noting that these cell wall-degrading enzymes do not act alone but require the synergistic action of multiple chitinases to effectively degrade fungal cell walls [53,54]. *Trichoderma* species are capable of secreting hydrolytic enzymes that specifically target and degrade the cell walls of host fungi [55]. For example, Chit62 inhibits pathogen growth by degrading chitin [56], while BsGlc157A glucanase disrupts cell wall integrity by hydrolyzing the glucan backbone [57]. Both chitinases and glucanases play important roles in this process [58]. The significant upregulation of these genes suggests that strain BBR–A enhances its ability to degrade the cell wall structure of the pathogen, providing a molecular basis for subsequent hyphal penetration and lysis, further confirming that this strain effectively inhibits *B. cinerea* growth through mycoparasitic activity. In addition to enzymatic degradation, antifungal secondary metabolites and volatile organic compounds also play important roles in the antagonistic system of *Trichoderma* [11,44]. The enrichment of secondary metabolism-related pathways in the transcriptome of this study suggests that such metabolites may participate in the synergistic inhibition of *B. cinerea* (Figure 2E), although their exact contributions require further metabolic and functional validation.

In terms of inducing plant resistance, this study revealed an antioxidant defense system driven by metabolic reprogramming through metabolomic and physiological biochemical analyses (Figure 5). Previous studies have shown that the upregulation of genes involved in caffeic acid biosynthesis can enhance antioxidant enzyme activity [59]. In the present study, the significant accumulation of caffeic acid metabolites coinciding with a marked increase in antioxidant enzyme activity suggests that BBR–A enhances the plant’s antioxidant capacity by promoting the synthesis of phenolic compounds. Phenylalanine metabolism is a key pathway for the synthesis of phenylpropanoids and defense-related secondary metabolites. Research has shown that plants can supplement phenylalanine metabolism via the phenylpyruvate pathway to enhance salicylic acid signaling and activate peroxidase gene expression [60]. Although direct phenylalanine metabolites were not detected in this study, the identification of phenylacetic acid derivatives and other precursor substances (Figure 5D) suggests that this pathway may have been partially activated. The salicylic acid and jasmonic acid signaling pathways play central roles in plant defense responses. Previous studies have demonstrated that *Trichoderma* can improve the rhizosphere microecology and regulate plant-microbe interactions [61], and its culture filtrate can induce systemic resistance by upregulating PR1-b gene expression [62]. The proteomic data from this study showed significant changes in hormone signaling and energy metabolism in blueberry leaves, and the enhanced hormone signaling further suggests that BBR–A may activate the immune network of blueberries through similar mechanisms.

*Trichoderma* can secrete microbe-associated molecular patterns (MAMPs) to activate the plant’s innate immune response and promote the accumulation of proline and soluble sugars, thereby enhancing the plant’s tolerance to abiotic stress [11]. In this study, significant changes in proteins related to amino acid and glycolipid metabolism (Figure 5E) indicate that metabolic reprogramming provides essential substrates and energy for the establishment of plant resistance. Furthermore, following treatment, the phenylpropane, flavonoid biosynthesis pathways, and ascorbate-glutathione cycle were significantly activated in blueberry leaves, leading to the accumulation of various phenolic derivatives and antioxidant metabolites (Figure 5E). These phenolic compounds can protect the antioxidant defense system by scavenging reactive oxygen/nitrogen species, inhibiting the activity of enzymes involved in free radical generation, or chelating trace metal ions [63]. This mechanism is consistent with the observed upregulation of caffeic acid metabolites and reduced H_2_O_2_ accumulation in this study (Figure 6A,B), further confirming the critical role of metabolic regulation in plant stress responses. Previous studies have shown that after pre-treatment with *T. asperellum*, only weak H_2_O_2_ accumulation was detected in infected leaves, indicating that this biocontrol agent promotes plant resistance by suppressing ROS formation [64,65]. According to research, *Trichoderma* can enhance the activity of key defense-related enzymes, such as phenylalanine ammonia-lyase (PAL), polyphenol oxidase (PPO), peroxidase (POD), and catalase (CAT), significantly reducing the lesion length of bacterial blight in rice [66]. Additionally, *T. asperellum* has been shown to inhibit the expansion of lesions caused by *Magnaporthe oryzae* and promote the activities of chitinase (CHI), β-glucosidase (GLU), lipoxygenase (LOX), and phenylalanine ammonia-lyase (PAL) [67]. However, in this study, only the activity of POD was verified. The activities of other antioxidant enzymes and the expression of related genes remain to be further investigated and will be the focus of future work. In summary, BBR–A enhances blueberry disease resistance by promoting the accumulation of antioxidant metabolites and activating the antioxidant enzyme system, forming an efficient reactive oxygen species (ROS) scavenging network that combines enzymatic and non-enzymatic mechanisms. This effectively reduces oxidative damage and maintains cellular redox homeostasis, representing one of the key molecular physiological mechanisms underlying its enhanced disease resistance.

## 5. Conclusions

In this study, *T. asperellum* BBR–A can effectively inhibit the growth and pathogenicity of *B. cinerea* BC7–1 through multiple mechanisms, including antagonism, mycoparasitism, and metabolic regulation. It also significantly delays disease development in blueberries and induces host resistance, indicating its strong potential as a biological control agent against blueberry gray mold.

## Data Availability

The transcriptome dataset is available in the CNCB Sequence Read Archive under accession number PRJCA064241 at https://ngdc.cncb.ac.cn/gsa/s/LfT0gwS0 (accessed on 13 May 2026), reference number [68]. The metabolomics dataset is available in the CNCB Sequence Read Archive under accession number PRJCA064282 at https://ngdc.cncb.ac.cn/omix/preview/CCBbxQZd (accessed on 20 May 2026), reference number [69]. The data that support the findings of this study are openly available on Figshare at https://doi.org/10.6084/m9.figshare.32532867 (accessed on 1 June 2026), reference number [70].

## Supplementary Materials

The following supporting information can be downloaded at: https://www.mdpi.com/article/10.3390/jof12070515/s1. Figure S1: Sequence alignment between T. asperellum BBR–A and B. cinerea BC7–1; Figure S2: qPCR validation of genes TrAFT101_011200, TrAFT101_004666, TrAFT101_010746, TrAFT101_006562, PRB1, and GNPDA1; Figure S3: Standard curves; Table S1: Primers for qPCR validation of 9 genes and actin primers.
