# Supplementary material for: Trichoderma asperellum Suppresses Gray Mold Caused by Botrytis cinerea and Enhances Disease Resistance of Blueberry
Source: J Fungi (Basel). 2026 Jul 14;12(7):515. doi: 10.3390/jof12070515 (PMC13412877; doi:10.3390/jof12070515)
Supplement: Supplementary file 1 [file jof-12-00515-s001.zip › jof-4382782-supplementary.pdf]

Table S1 Primers for qPCR validation of 9 genes and actin primers.

Gene	Primer
CBH1	F: ATGATTACGCTGCCAATATG<br>R: GTGCTACCAATAGGTCCAA
GNPDA1	F: AACCGTATCAACACCTTCC<br>R: ACATTCTCGAATGAGACCAG
TrAFT101_010746	F: TGACTGTTGCTGTGATGAT<br>R: AATGATGACGGCGACAAG
TrAFT101_004666	F: AAGCCGCCATTGATAATAAC<br>R: CAAGCCCGAGAGTAACAG
CHIT37	F: TTGCTCTGGCGACTCTTA<br>R: CGTATGGAACCTTGATTGTG
CHIT46	F: CACTATTCTGACGATTCCTG<br>R: GAGAGCATAACCTTCAAGTTG
PRB1	F: GATGTTGCGTCTGTGGAG<br>R: CTGATGGGAGGTGTTGATAC
TrAFT101_006562	F: GCAACAGATTCAACCTCTC<br>R: GATGTATAATAGCACCAGGATC
TrAFT101_011200	F: AATGGAGTGTCTTTGAGGAG<br>R: ACTACAGCCAGTTGAATGAT
TrAFT101_008842	Actin-F: AGTCCAACCGTGAGAAGA<br>Actin-R: CAATGGCGTGAGGAAGAG

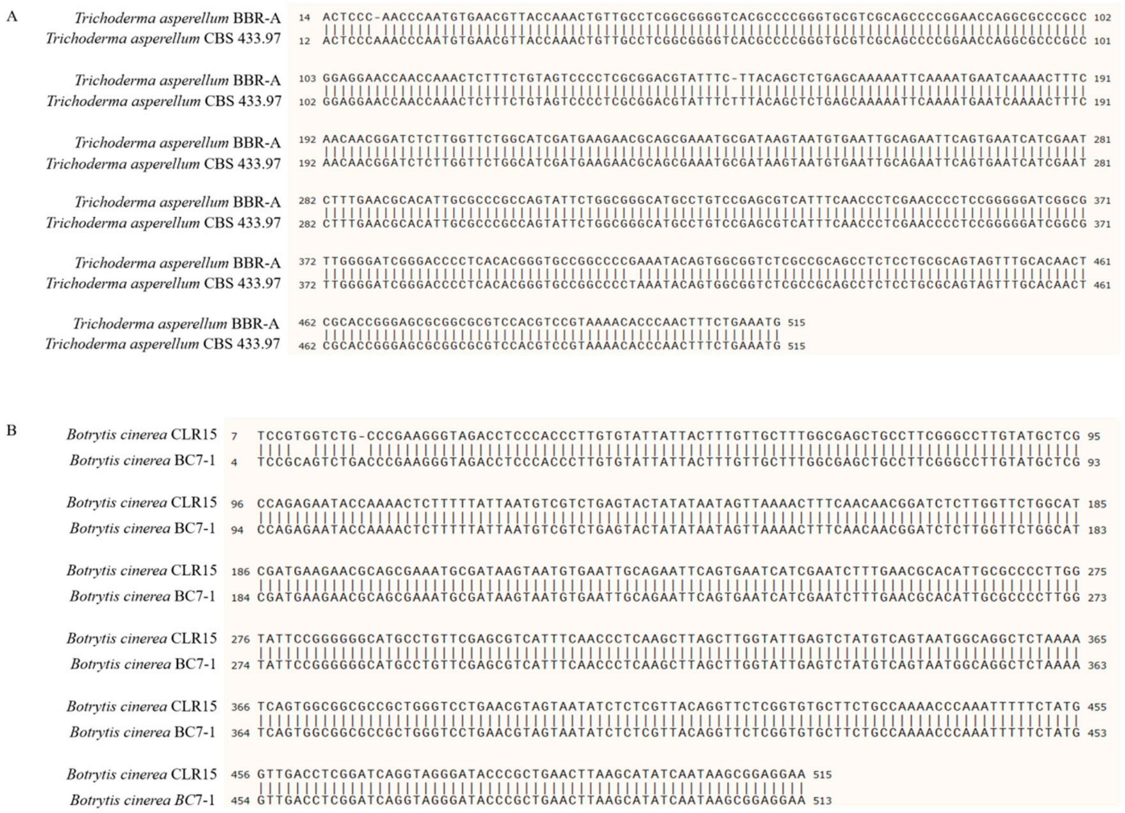

Figure S1. Sequence alignment between *T. asperellum* BBR-A and *B. cinerea* BC7-1. (A) Sequence alignment diagram of *T. asperellum.* (B) Sequence alignment diagram of *B. cinerea.*

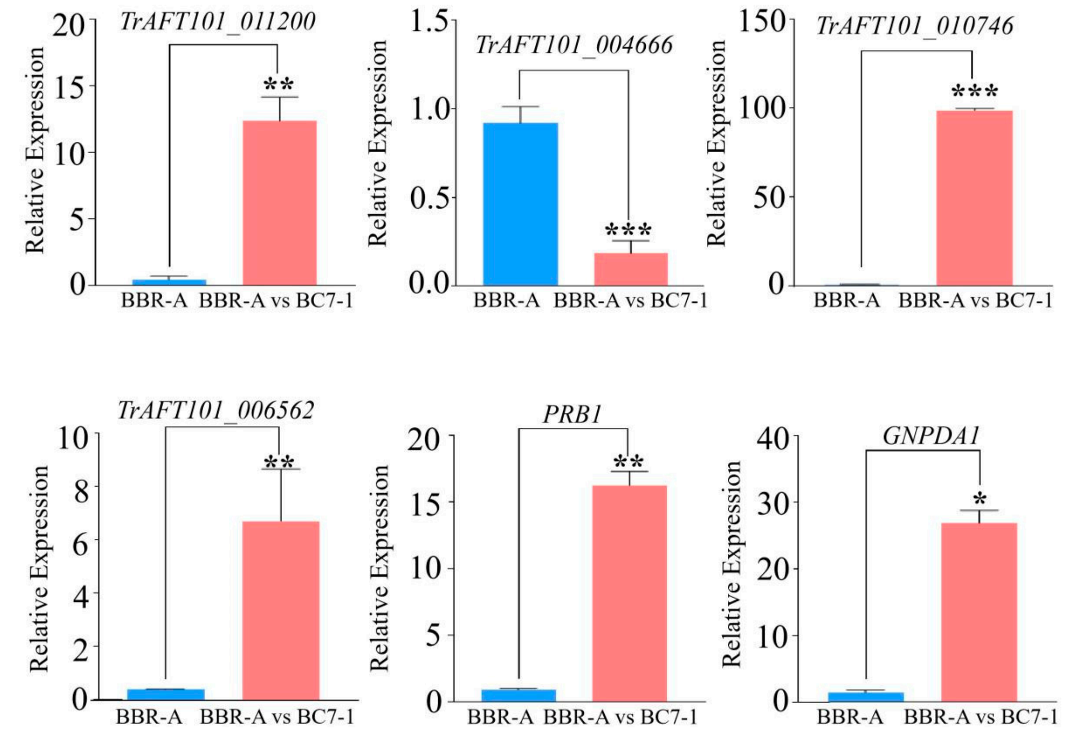

Figure S2. qPCR validation of genes *TrAFT101_011200*, *TrAFT101_004666*, *TrAFT101_010746*, *TrAFT101_006562*, *PRB1*, and *GNPDA1*. Error bars represent mean ± SD from three replicates. ns, $p > 0.05$; *$p < 0.05$, **$p < 0.01$, ***$p < 0.001$.

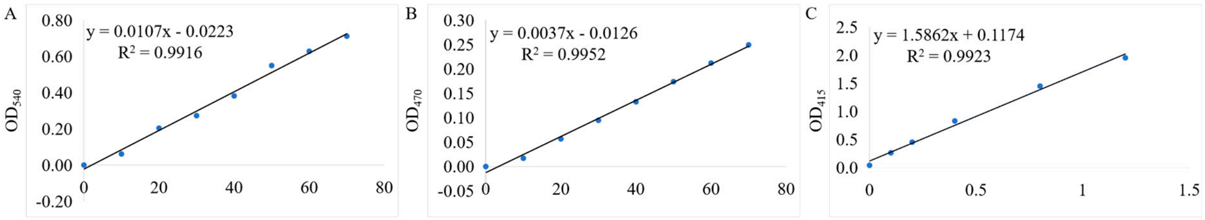

Figure S3. Standard curves. (A) Cellulose standard curve. (B) Chitin standard curve. (C) H_2O_2 standard curve.
